# The persuasive effects of political microtargeting in the age of generative artificial intelligence

**DOI:** 10.1093/pnasnexus/pgae035

**Published:** 2024-01-29

**Authors:** Almog Simchon, Matthew Edwards, Stephan Lewandowsky

**Affiliations:** School of Psychological Science, University of Bristol, Bristol BS8 1QU, UK; Department of Psychology, Ben-Gurion University of the Negev, Beer Sheva 841050, Israel; Department of Computer Science, University of Bristol, Bristol BS8 1QU, UK; School of Psychological Science, University of Bristol, Bristol BS8 1QU, UK; School of Psychological Science, University of Western Australia, Perth 6009, Australia; Department of Psychology, University of Potsdam, Potsdam 14476, Germany

**Keywords:** microtargeting, persuasion, GPT, AI

## Abstract

The increasing availability of microtargeted advertising and the accessibility of generative artificial intelligence (AI) tools, such as ChatGPT, have raised concerns about the potential misuse of large language models in scaling microtargeting efforts for political purposes. Recent technological advancements, involving generative AI and personality inference from consumed text, can potentially create a highly scalable “manipulation machine” that targets individuals based on their unique vulnerabilities without requiring human input. This paper presents four studies examining the effectiveness of this putative “manipulation machine.” The results demonstrate that personalized political ads tailored to individuals’ personalities are more effective than nonpersonalized ads (studies 1a and 1b). Additionally, we showcase the feasibility of automatically generating and validating these personalized ads on a large scale (studies 2a and 2b). These findings highlight the potential risks of utilizing AI and microtargeting to craft political messages that resonate with individuals based on their personality traits. This should be an area of concern to ethicists and policy makers.

**Significance Statement**

Our study sheds light on the impact of artificial intelligence (AI) in political advertising, revealing how automated, personalized messages can potentially shape voter decisions. Our findings, showcasing the power and scalability of this technology, underscore the pressing need for ethical scrutiny and policy-oriented solutions to govern the use of AI in shaping public opinion and safeguarding electoral integrity.

## Introduction

In the summer of 2016, the world was struck with the Brexit referendum results, in which the United Kingdom had voted to leave the European Union. On the other side of the Atlantic, Donald Trump was only months away from getting elected to be the 45th president of the United States. At the same time, Alexander Nix, a CEO of a relatively obscure company called Cambridge Analytica, promoted the company’s ostensible success at swaying voters from one political candidate to another based on exploitation of voters’ particular psychological vulnerabilities. This tactic involves deducing psychological attributes that are not readily observable, such as personality traits, from individuals’ online behavior and personal data. Subsequently, these inferred psychological features are leveraged to craft highly personalized messages tailored to each individual. Cambridge Analytica was involved in the Vote Leave campaign (United Kingdom), the 2016 Trump campaign (United States), and other political campaigns spanning 68 countries (1) before it folded in 2018 after investigations opened in several countries. The investigation by a British Parliamentary committee concluded that relentless targeting that plays “to the fears and the prejudices of people, in order to alter their voting plans” is “more invasive than obviously false information” and contributes to a “democratic crisis” (2). Microtargeting is also problematic outside the political domain when it exploits people’s moment-to-moment emotional state. Facebook has access to technology that can identify vulnerable teenagers at moments when they feel “worthless” and “insecure,” although the technology was ostensibly never made available to advertisers and only used in an experimental context (3).

The actual impact of Cambridge Analytica is difficult to ascertain. There is, however, little doubt that sensitive personal characteristics can be inferred from people’s digital fingerprint. A review of 327 studies concluded that numerous demographics could be reliably inferred from personal data, including for example sexual orientation (4). Personality (specifically, the “Big 5” attributes) can be inferred from 300 Facebook likes with greater accuracy than a person’s own spouse (5).

It is less clear that microtargeting is indeed effective as a persuasion tool. The claims initially advanced by Cambridge Analytica were most likely exaggerated (6), and some experiments have failed to find a benefit from personalizing and microtargeting political ads in comparison to exposure to a generic ad that is the same for all members of the target audience (7). By contrast, a recent set of studies has repeatedly shown the efficacy of microtargeting in a variety of circumstances. For example, microtargeting can increase voter turnout during tight political competitions based on highly salient issues (8); it can prevent voter defection from a party they initially favored (9); and it can have an effect even when ads are targeted on the basis of a single personal attribute (10). A recent systematic review established that message-tailoring (i.e. aligning messages with characteristics of the target audience) is an effective persuasion strategy ( r=0.17) (11).

Given that, of late, the evidence has tilted in favor of the efficacy of microtargeting, the availability of generative artificial intelligence (AI) (e.g. ChatGPT) has triggered additional concern that those large language models (LLMs) could be leveraged to readily scale microtargeting. Whereas conventional ad production is a time-consuming process and personalization requires painstaking validation (12), two recent technological developments can dramatically reduce the time and effort required to construct ads: First, generative AI may be used to derive numerous personalized versions of political messages from a single human-designed template. Second, a recent machine-learning model has successfully inferred the personality of readers from the text they consume (13), providing a platform for rapid validation of the text produced by the AI. The technology thus exists to take any political message and derive versions that target people of different personalities in an automated manner and at scale.

The present paper tests the effectiveness of this “manipulation machine” in four studies. We show that personality-congruent political ads are indeed more effective than other ads (studies 1a and 1b), and we show that ads can be generated and validated automatically (studies 2a and 2b).

## Results

In study 1, we explored the effect of personality-congruent political messages on perceived persuasion using real political ads. We selected 10 ads from a pool of 1,552 political ads that were published on Facebook to UK users between December 2019 and December 2021 (for validation, see Ref. (13)). Leveraging a recent language model, we assigned an “openness score” to each of these ads, predicting the attractiveness of the ad’s text for the openness to experience personality dimension experience (13).^a^ In study 1a, we collected data from 440 participants; in study 1b, we replicated the procedure with 804 participants. Participants were requested to rate the ads’ perceived persuasiveness (see Materials and methods for details) and answer a personality questionnaire measuring the “openness to experience” factor (14). We constructed a personality matching score, wherein we calculate the absolute difference between the scaled openness score of participant *i* and the scaled openness score of ad *j*: ${\text{Matching}}_{ij}=|z({\text{Openness}}_{i})-z({\text{Openness}}_{j})|$, which serves as our key predictor of perceived persuasion.

**Fig. 1.**
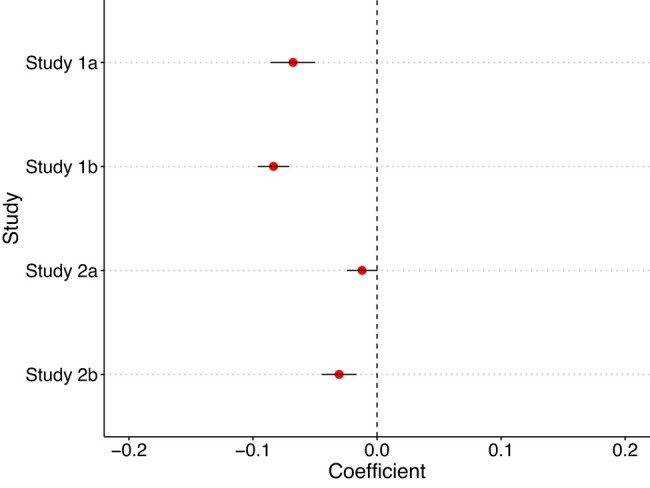
Perceived persuasion effect in studies 1a (Facebook ads; n=440), 1b (Facebook ads; n=803), 2a (GPT-3; n=803), and 2b (ChatGPT; n=804). *x*-Axis denotes the matching score coefficients. Negative coefficients mean that the larger the deviance from personality matching, the less persuasive the message was rated. *y*-Axis denotes the different studies. Error bars indicate 95% CI. The dashed line represents the zero point.

We fitted a linear mixed model to predict perceived persuasion with the openness score of the person, openness score of the ad and matching score as predictors. The model included random intercepts for ads and participants. The matching score was a significant predictor of perceived persuasion, above and beyond the personality score of people and ads, such that the larger the deviance from matching, the less persuasive the message was rated. Matching effect—study 1a: b=−0.07,95% CI ( −0.09, −0.05), t(26,348)=−7.40, P<0.001; matching effect—study 1b: b=−0.08, 95% CI ( −0.10, −0.07), t(48173)=−12.87, P<0.001, see Fig. 1 and Supplementary material for details.

Next, we explored whether this process could be automated using generative AI. We leveraged two generative language models: GPT-3 and ChatGPT. We prompted the models with instructions to rephrase the existing ads in our ad corpus and generate two variations of each ad, one catering to people high on openness and one catering to people low on openness (see Materials and methods for details). It is important to recognize that our approach is limited to a single iteration of the generated ad. Given the stochastic nature of LLMs, some outputs may be better than others. However, our argument is that LLMs could potentially be used to produce manipulative microtargeting at scale. Consequently, the minor variations introduced by these random fluctuations are likely inconsequential.

To ensure that the text generated by the language models appeals to different personality types, we applied our predictive model (13) and assigned an “openness score” to each ad in the generated corpora. In both corpora, we found evidence that the prompts led to generating ads in the expected direction, e.g. higher openness scores with high-openness prompts). Study 2a: t(870)=2.07, P=0.039, Cohen’s d=0.07, 95% CI (0.00, 0.14); study 2b: t(995)=16.01, P<0.001, Cohen’s d=0.51, 95% CI (0.44, 0.57). In addition to the current algorithmic validation, we also validated a smaller sample using human raters; see Supplementary material for details.

We then selected 10 ad pairs from each model that maximized the difference between the members of each pair (see Supplementary material) and ran the same experimental pipeline that was used for study 1 ( $n_{2a}=803$, $n_{2b}=804$).

In study 2a (GPT-3), the matching score narrowly failed to reach statistical significance: b=−0.01,95% CI ( −0.02, 3.43 e−04), t(48173)=−1.91, P=0.057. However, the effect was statistically significant with the more powerful language model (study 2b; ChatGPT). Matching effect: b=−0.03, 95% CI ( −0.04, −0.02), t(48233)=−4.25, P<0.001, see Fig. 1 and Supplementary material for details.

## Discussion

Our findings indicate that political microtargeting is an effective technique and can be automated using off-the-self generative AI. While we show consistent efficacy across four studies, it is important to recognize that the demonstrated effect sizes are rather small. Nonetheless, small effect sizes can turn substantial at scale, and the automation of political microtargeting is pivotal to achieving such scale. To put this effect size in context, based on the change between the median mismatch and no mismatch between individuals and ads, we have simulated 100,000 responses. Given a cutoff of 3 (midpoint of the 5-point perceived persuasion scale), we find that out of every 100,000 individuals exposed to political messages tailored to their personality, 2,490 individuals would now be expected to be persuaded due to the style. By extrapolation, the change from the worst matching to the best matching would mean an increase to 11,405 individuals. Considering that elections are often decided by fractions of a percentage point, shifting a few thousand individuals out of 100,000 can substantially impact the results.

One notable drawback of utilizing OpenAI products (such as ChatGPT) is their closed-source nature. In response to this concern, we conducted an additional algorithmic validation of the stimuli. We replicated our algorithmic validation using an open-source model (Meta’s Llama 2 70-B), consistently reaching similar conclusions (see Supplementary material for more details).

Our study focused on the openness factor of personality as a test case, yet recent evidence with consumer products suggests that persuasive microtargeted messages generated by LLMs can go beyond targeting openness. There is also evidence that microtargeting can be successfully applied to moral reframing of political texts (e.g. appealing to fairness or loyalty when advocating for climate action) (15).

Unlike other malicious uses of ChatGPT, such as generating fraudulent emails or phishing websites, rephrasing inputs in a microtargeting manner does not violate OpenAI’s usage policies, and there are accordingly no built-in safeguards to prevent this usage of the model. Advertisers, political campaign managers, and all manner of interest groups currently have access to this technology. In one sense, this is a democratization of capability, as anyone may create targeted content. However, the benefits of targeting would be expected to accrue to those actors who are best-placed to deliver politically targeted content at scale to very large populations. And there is nothing to stop those actors to devise content that is untruthful or manipulative or both, creating the specter of “gaslighting” populations by exploiting individual vulnerabilities.

In light of the potential harm of large-scale microtargeted manipulation, one might be concerned that discussing and researching manipulative strategies, as we did here, might inadvertently promote their use, potentially causing more harm. However, we believe that providing scientific evidence is essential for regulators, policymakers, and the public to make well-informed decisions about how to contain such risks. Although the possibility exists that malicious actors could misuse our research, we argue that the benefits of contributing solid, science-backed insights to the public discourse far outweigh this risk.

Previous research has demonstrated that it is possible to detect microtargeting efforts automatically (13). Our current findings further support the notion that these efforts are not only detectable but also effective and scalable. Given this understanding, there is a need for behavioral and cognitive science to concentrate on developing prevention methods through the design of interventions that enhance people’s ability to detect manipulation efforts and make informed decisions in their online environments.

The effectiveness of advertisements can significantly diminish when individuals become aware of unacceptable targeting practices, such as using external data or inferred information without their consent (16). Hence, one potential intervention could focus on informing people when microtargeting takes place. Leveraging a predictive model, e.g. (13), instances where personality matching appears suspiciously accurate could be identified, which could then flag potential manipulation to the user. The proposed intervention would promote transparency and user-agency by alerting users when ads seem “too good to be true” in terms of personality match. Recent evidence suggests that simply providing information about one’s personality can be an effective boosting technique in detecting microtargeted messages (17). Building upon this, we lay the groundwork for an extended intervention that combines information from both the individual and the ad, creating a more comprehensive approach that can be applied in ecological settings.

While enhancing microtargeting awareness is valuable, it alone cannot fully address the challenges stemming from information and power imbalances between platforms and users. To promote a fair and equitable digital landscape, it is essential to design an internet for democracy, prioritizing transparency and user empowerment over profit-driven corporations (18).

## Materials and methods

Participants were recruited from a UK-based prolific sample. Study 1a: n=440, $M_{\mathrm{age}}=39.45$, $SD_{\mathrm{age}}=12.58$; study 1b: n=804, $M_{\mathrm{age}}=40.58$, ${\text{SD}}_{\mathrm{age}}=13.04$; study 2a: n=804, $M_{\mathrm{age}}=41.76$, $SD_{\mathrm{age}}=13.69$, study 2b: n=803, $M_{\mathrm{age}}=40.59$, $SD_{\mathrm{age}}=13.62$.

The studies lasted for approximately 6 min, and participants received £1.8 in study 1a and £0.90 in studies 1b, 2a, and 2b as compensation. All studies were fully reviewed and approved by the School of Psychological Science Research Ethics Committee at the University of Bristol (ethics approval #12318 and #12883). All participants provided informed consent via mouse click prior to their participation.

In all studies, participants were presented with 10 ads in a Facebook ad format. For each ad, they were requested to rate the perceived persuasiveness of the ad (on a 1–5 Likert scale), measured with six items adapted from Ref. (19) (see Supplementary material for details). Scholars have questioned the use of self-reports for studying persuasion effects and suggested using tangible behaviors like click-through rates (CTRs) instead (12). However, whereas CTR is appropriate for studying consumer behavior, our focus is on endorsement of ideas and ideologies. In this attitudinal playing field, self-reports have a strong track record in microtargeted persuasion (15, 19). A meta-analysis revealed a reliable link between self-reported persuasion and measures of attitudes or intentions ( r=0.41) (20), confirming the appropriateness of perceived persuasion ratings for our research. In studies 2a and 2b, there were two variations of each ad; therefore, participants were randomly assigned to either a low-openness or high-openness version of each ad (10 ads altogether out of 20). At the end of the survey, participants were requested to answer 12 items of the openness to experience personality factor, measured by BFI-2 (14) on a 1–5 Likert scale.

### Prompting strategy

We followed a specific process to create two versions of each ad in study 2. First, we provided GPT-3 and ChatGPT with the definition of openness to experience:

One way in which people differ from each other is through their personalities. The Big-Five Personality Model identifies five personality traits on which each individual varies. One of the traits is Openness to Experience. Openness to Experience has both motivational and structural components. People high in Openness to Experience are motivated to seek new experiences and to engage in self-examination. People low in Openness to Experience by contrast are more comfortable with familiar and traditional experiences.

Then, we made a request in the following manner:

Please rephrase the next ad so it would appeal to people high/low on Openness to experience.

In our ChatGPT (gpt-3.5-turbo; February 2023) prompt, we included the additional instruction:

Do not oversell, but make it slightly more persuasive to people who are high/low on Openness to experience.

As a result, each prompt was constructed in a structured order: openness definition, instruction, and the original ad. See Supplementary material for more details.

### Open science statement

Studies 1a, 2b, and 2a were preregistered at https://aspredicted.org/4Q1_LTB, https://aspredicted.org/XWR_3JS, and https://aspredicted.org/PZ1_K5B, respectively. Study 2b was not preregistered. Our hypotheses and target sample size remained untouched. However, the preregistered analysis plan relied on binary classification of the ads. We deviated from this plan and incorporated a continuous measure of ad openness, which allowed us to simplify the model and focus on the matching score metric. In the Supplementary material, we report all the preregistered analyses and show overall convergence with the current method. Full reproduction materials are accessible at https://osf.io/5w3ct.

## Supplementary Material

pgae035_Supplementary_Data

## Data Availability

All shareable data and code are available at https://osf.io/5w3ct.

## Note

a An error was identified in the design, where one of the ads appeared twice instead of another ad. As a result, the findings of study 1a are limited to a set of nine ads. This issue was rectified in study 1b.

## Preprints

This manuscript was posted on a preprint https://doi.org/10.31234/osf.io/62kxq.
