# Supplementary material for: The persuasive effects of political microtargeting in the age of generative artificial intelligence
Source: PNAS Nexus. 2024 Jan 29;3(2):pgae035. doi: 10.1093/pnasnexus/pgae035 (PMC10849795; doi:10.1093/pnasnexus/pgae035)
Supplement: pgae035_Supplementary_Data [file pgae035_supplementary_data.pdf]

Supplementary Information for

The persuasive effects of political microtargeting in the age of generative AI

Almog Simchon, Matthew Edwards, and Stephan Lewandowsky

almogsi@post.bgu.ac.il

This PDF file includes:

Supplementary text
SI References

Supporting Information Text

Extended methods.

Adapted ad perceived persuasiveness scale. In order to measure perceived persuasion, we adapted the ad perceived persuasiveness scale (1) to include the following six items: 1. "I find this ad to be persuasive"; 2. "This is an effective ad"; 3. "I would click on this link after seeing this ad"; 4. "Overall, I like this ad"; 5. "This ad has made me more interested in the topic"; 6. "I am interested in learning more about this topic after seeing this ad". Participants rated the effectiveness of each ad on a 5-point Likert scale ranging from "strongly disagree" to "strongly agree".

Psychometric evaluation of the scale (based on Study 1b) suggests the scale has high internal consistency. A multilevel-reliability analysis (2) indicated a multilevel Cronbach's alpha of 0.93. An exploratory factor analysis suggests that a single factor best explains the variance structure of the items.

Ad curation and human validation. The ad corpus was described in (3) and included 1552 political ads that were published on Facebook to UK users between December 2019 and December 2021. The stimulus set used in Studies 2a and 2b was a subset of those validated in (3).

In Studies 2a and 2b, the stimuli were designed to maximize the difference score between pairs of ads. As ads were generated by GPT-3 and ChatGPT, each variant received a score based on our internal predictive model. We calculated the absolute difference between the predictions for members of each pair and selected 30 ads in each study that received the highest absolute difference. We then selected 24 ad-pairs to be validated in Study 2a and 17 ad-pairs in Study 2b.

In all final stimulus sets, references to unions, parties, politicians, or other individuals were replaced with a generic tag (e.g., "no more broken promises, no more lies! #TimeToGo[POLITICIAN]")

Ethics declaration All studies were fully reviewed by the School of Psychological Science Research Ethics Committee at the University of Bristol (ethics approval #12318 and #12883). All participants provided informed consent via mouse click prior to their participation.

Study 2a validation We recruited 100 participants from a UK Prolific sample. The study lasted for approximately 6 minutes, and participants received £1.50 as compensation.

The validation studies were approved by the Research Ethics Committee of the School of Psychological Science at the University of Bristol (approval code 12318).

Participants received the following instructions:

> Please spend some time reading the information on this page. Then press the button below to start the experiment.
>
> One way in which people differ from each other is through their personalities. The Big-Five Personality Model identifies five personality traits on which each individual varies. One of the traits is Openness to Experience.
>
> Openness to experience has both motivational and structural components. People high in openness are motivated to seek new experiences and to engage in self-examination. Closed people by contrast are more comfortable with familiar and traditional experiences.
>
> In the following task you will be presented with a series of political advertisements that have been published in the UK over the last 5 years. For each advertisement, you will be asked to identify to what extent each advertisement is oriented towards people on the Openness to experience scale, ranging from Very Closed (lowest) to Very Open (highest).
>
> Please press the button below to start the experiment.

Then, participants were presented with one variant of each ad (either open or closed), and they were requested to rate it on a 1-6 Likert scale ranging from "Strongly Close to experience" to "Strongly Open to experience.

We performed a t-test per each ad pair, correcting for multiple comparisons (Bonferroni correction). Only two ads did not reach statistical significance (corrected p values = 0.057 and 0.051). Effect sizes ranged between 0.39 to 2.95. For full results, see the OSF repository.

Study 2b validation We recruited 50 participants from a UK Prolific sample. The study lasted for approximately 5 minutes, and participants received £0.90 as compensation. We followed the same pipeline as in the validation of Study 2a.

Here again, we performed a t-test per each ad pair, correcting for multiple comparisons (Bonferroni correction). Just two ads did not reach statistical significance (corrected p values = 0.923 and 0.067). Effect sizes ranged between -0.02 to 2.07. For full results, see the OSF repository.

Full regression results. The main text includes linear mixed models results for our main metric, the matching score. Here we report full regression results that include all the effects.

We follow the following notations: Scaled openness score of participant $i = z(openness_i)$; the scaled openness score of ad j $= z(openness_j)$; $matching_{ij} = |z(openness_i) - z(openness_j)|$.

Study 1a We fitted a linear mixed model (estimated in R using REML and *nloptwrap* optimizer) to predict perceived persuasion with $z(openness_j)$, $z(openness_i)$, $matching_{ij}$ (formula: value $\sim z(openness_j) + z(openness_i) + matching_{ij}$). This modeling procedure holds for all studies. The model included random intercepts (formula: list($\sim 1 \mid ad_j$, $\sim 1 \mid participant_i$)).

The model's total explanatory power is substantial (conditional $R^2 = 0.28$), and the part related to the fixed effects alone (marginal R^2) is of 8.63e-03. The model's intercept, corresponding to $z(openness_j) = 0$, $z(openness_i) = 0$ and $matching_{ij} = 0$, is at 2.79 (95% CI [2.65, 2.94], $t(26348) = 37.13$, $p < .001$). Within this model the effect of $z(openness_j)$ is statistically non-significant and negative (b = -0.01, 95% CI [-0.15, 0.12], $t(26348) = -0.18$, $p = 0.854$; Std. β = -0.01, 95% CI [-0.12, 0.10]). The effect of $z(openness_i)$ is statistically significant and positive (b = 0.10, 95% CI [0.04, 0.16], $t(26348) = 3.16$, $p = 0.002$; Std. β = 0.08, 95% CI [0.03, 0.13]). The effect of $matching_{ij}$ is statistically significant and negative (b = -0.07, 95% CI [-0.09, -0.05], $t(26348) = -7.40$, $p < .001$; Std. β = -0.04, 95% CI [-0.06, -0.03]).

Study 1b The model's total explanatory power is substantial (conditional $R^2 = 0.31$), and the part related to the fixed effects alone (marginal R^2) is of 8.31e-03. The model's intercept, corresponding to $z(openness_j) = 0$, $z(openness_i) = 0$ and $matching_{ij} = 0$, is at 2.73 (95% CI [2.60, 2.86], $t(48173) = 41.79$, $p < .001$). Within this model the effect of $z(openness_j)$ is statistically non-significant and positive (b = 0.06, 95% CI [-0.06, 0.18], $t(48173) = 1.02$, $p = 0.310$; Std. β = 0.05, 95% CI [-0.05, 0.15]). The effect of $z(openness_i)$ is statistically significant and positive (b = 0.06, 95% CI [8.98e-03, 0.10], $t(48173) = 2.34$, $p = 0.019$; Std. β = 0.04, 95% CI [7.22e-03, 0.08]). The effect of $matching_{ij}$ is statistically significant and negative (b = -0.08, 95% CI [-0.10, -0.07], $t(48173) = -12.87$, $p < .001$; Std. β = -0.05, 95% CI [-0.06, -0.05]).

Study 2a The model's total explanatory power is substantial (conditional $R^2 = 0.43$), and the part related to the fixed effects alone (marginal R^2) is of 7.45e-03. The model's intercept, corresponding to $z(openness_j) = 0$, $z(openness_i) = 0$ and $matching_{ij} = 0$, is at 2.51 (95% CI [2.29, 2.72], $t(48173) = 22.88$, $p < .001$). Within this model the effect of $z(openness_j)$ is statistically non-significant and positive (beta = 0.10, 95% CI [-0.11, 0.31], $t(48173) = 0.97$, $p = 0.331$; Std. beta = 0.09, 95% CI [-0.09, 0.26]). The effect of $z(openness_i)$ is statistically non-significant and negative (beta = -0.01, 95% CI [-0.06, 0.03], $t(48173) = -0.62$, $p = 0.536$; Std. beta = -0.01, 95% CI [-0.05, 0.03]). The effect of $matching_{ij}$ is statistically non-significant and negative (beta = -0.01, 95% CI [-0.02, 3.43e-04], $t(48173) = -1.91$, $p = 0.057$; Std. beta = -8.11e-03, 95% CI [-0.02, 2.31e-04]).

Study 2b The model's total explanatory power is substantial (conditional $R^2 = 0.32$), and the part related to the fixed effects alone (marginal R^2) is of 0.01. The model's intercept, corresponding to $z(openness_j) = 0$, $z(openness_i) = 0$ and $matching_{ij} = 0$, is at 2.85 (95% CI [2.71, 2.98], $t(48233) = 40.03$, $p < .001$). Within this model the effect of $z(openness_j)$ is statistically non-significant and negative (b = -0.12, 95% CI [-0.25, 0.01], $t(48233) = -1.75$, $p = 0.080$; Std. β = -0.10, 95% CI [-0.20, 0.01]). The effect of $z(openness_i)$ is statistically non-significant and positive (b = 0.03, 95% CI [-9.78e-03, 0.08], $t(48233) = 1.53$, $p = 0.127$; Std. β = 0.03, 95% CI [-7.93e-03, 0.06]). The effect of $matching_{ij}$ is statistically significant and negative (b = -0.03, 95% CI [-0.04, -0.02], $t(48233) = -4.25$, $p < .001$; Std. β = -0.02, 95% CI [-0.03, -0.01].

Pre-registered analyses. Our final modeling approach differed from the one listed in our pre-registrations because in retrospect, the pre-registered analyses seemed suboptimal because dichotomizing a continuous variable seemed unnecessary and inadvisable. For full transparency, here we report the the original pre-registered models.

Study 1a We follow the following notations: openness score of participant $i = openness_i$; ad category $j = category_j$ (open/closed); interaction of $openness_i * category_j$ which serves as the main metric of matching. Note that in contrast to the main analysis, here we dichotomized the openness of the ad into high or low.

We fitted a linear mixed model (estimated using REML and BOBYQA optimizer) to predict perceived persuasion with *category* and *openness* (formula: $\hat{y} = category * openness$). The model included *category* as a random effect by participants, and *openness* as a random effect by ads (formula: list($\sim category \mid participant_i$, $\sim openness \mid ad_j$)). The model's total explanatory power is substantial (conditional $R^2 = 0.38$) and the part related to the fixed effects alone (marginal R^2) is of 7.14e-03. The model's intercept, corresponding to *category* = closed and *openness* = 0, is at 2.42 (95% CI [1.88, 2.96], $t(26344) = 8.77$, $p < .001$). Within this model: The effect of *category* [open] is statistically non-significant and negative (b = -0.43, 95% CI [-1.07, 0.21], $t(26344) = -1.31$, $p = 0.192$; Std. β = 0.03, 95% CI [-0.19, 0.25]). The effect of *openness* is statistically non-significant and positive (b = 0.07, 95% CI [-0.06, 0.21], $t(26344) = 1.10$, $p = 0.271$; Std. β = 0.04, 95% CI [-0.03, 0.11]). The effect of *category* [open] × *openness* is statistically non-significant and positive (b = 0.12, 95% CI [-0.03, 0.28], $t(26344) = 1.60$, $p = 0.110$; Std. β = 0.06, 95% CI [-0.01, 0.14]).

Study 1b We followed the same analytical procedure as Study 1a. The model's total explanatory power is substantial (conditional $R^2 = 0.41$) and the part related to the fixed effects alone (marginal R^2) is of 9.00e-03. The model's intercept, corresponding to *category* = closed and *openness* = 0, is at 2.55 (95% CI [2.19, 2.92], $t(48169) = 13.69$, $p < .001$). Within this model: The effect of *category* [open] is statistically significant and negative (b = -0.54, 95% CI [-0.97, -0.12], $t(48169) = -2.49$, $p = 0.013$; Std. β = 0.12, 95% CI [-0.06, 0.31]). The effect of *openness* is statistically non-significant and positive (b = 1.01e-03, 95% CI [-0.09, 0.09], $t(48169) = 0.02$, $p = 0.983$; Std. β = 5.50e-04, 95% CI [-0.05, 0.05]). The effect of *category* [open] × *openness* is statistically significant and positive (b = 0.19, 95% CI [0.08, 0.29], $t(48169) = 3.50$, $p < .001$; Std. β = 0.10, 95% CI [0.04, 0.16]).

Study 2b In the current study, we introduced more complexity, as pairs of ads needed to be modeled in a way that takes into account their dependence.

We fitted a linear mixed model (estimated using REML and BOBYQA optimizer) to predict perceived persuasion with *category* and *openness* (formula: $\hat{y} = type * openness$). The model included *category* as a random effect by participants *openness* as a random effect by ads The model included *category* as random effects (formula: list($category \sim participant_i$, $\sim category * openness \mid ad_j$, $\sim openness \mid ad_j * category_j$)). The model's explanatory power related to the fixed effects alone (marginal R^2; conditional R^2 could not be computed) is 0.02. The model's intercept, corresponding to $category$ = closed and $openness = 0$, is at 2.50 (95% CI [2.08, 2.92], $t(48159) = 11.66$, $p < .001$). Within this model: the effect of *category* [open] is statistically non-significant and positive ($b = 0.12$, 95% CI [-0.21, 0.46], $t(48159) = 0.74$, $p = 0.461$; Std. $\beta = 0.22$, 95% CI [-0.04, 0.49]). The effect of *openness* is statistically non-significant and negative (b = -0.04, 95% CI [-0.11, 0.04], $t(48159) = -0.99$, $p = 0.324$; Std. β = -0.02, 95% CI [-0.06, 0.02]). The effect of *category* [open] × *openness* is statistically non-significant and positive ($b = 0.04$, 95% CI [-0.04, 0.11], $t(48159) = 1.03$, $p = 0.305$; Std. $\beta = 0.02$, 95% CI [-0.02, 0.06]).

Study 2b This study was not pre-registered, but we reported it in the same way for completeness. The model's total explanatory power is substantial (conditional $R^2 = 0.37$) and the part related to the fixed effects alone (marginal R^2) is of 1.71e-03. The model's intercept, corresponding to $category$ = closed and $openness = 0$, is at 2.49 (95% CI [2.06, 2.93], $t(48219) = 11.30$, $p < .001$). Within this model: the effect of *category* [open] is statistically non-significant and positive ($b = 0.18$, 95% CI [-0.10, 0.46], $t(48219) = 1.24$, $p = 0.215$; Std. $\beta = 0.05$, 95% CI [-0.14, 0.24]). The effect of *openness* is statistically non-significant and positive ($b = 0.08$, 95% CI [-0.01, 0.17], $t(48219) = 1.70$, $p = 0.090$; Std. $\beta = 0.04$, 95% CI [-6.38e-03, 0.09]). The effect of *category* [open] × *openness* is statistically non-significant and negative (b = -0.03, 95% CI [-0.12, 0.06], $t(48219) = -0.68$, $p = 0.496$; Std. β = -0.02, 95% CI [-0.06, 0.03]).

Mini meta-analysis We conducted an internal meta-analysis of the interaction effect to assess the overall effect of personality and ad matching. In a fixed-effect meta-analysis we find evidence that the overall effect supports our pre-registered hypothesis and aligns with the analyses reported in the main text: estimate = 0.074, 95% CI [0.024, 0.124]), $z = 2.892$, $p = 0.004$.

Robustness checks. To assess the robustness of our method, we opted to assign openness ratings of ads through human feedback (see "Human validation" section) instead of relying on algorithmically derived openness scores as reported in the main text. Here we present an identical procedure to the one outlined in the full regression results section, with the primary distinction lying in the origin of the ad openness ratings (human instead of AI).

Study 1a We fitted a linear mixed model (estimated using REML and *nloptwrap* optimizer) to predict perceived persuasion with $z(openness_j)$, $z(openness_i)$, $matching_{ij}$ (formula: value $\sim z(openness_j) + z(openness_i) + matching_{ij}$). This modeling procedure holds for all studies. The model included random intercepts (formula: list($\sim 1 \mid ad_j$, $\sim 1 \mid participant_i$)). The model's total explanatory power is substantial (conditional $R^2 = 0.28$), and the part related to the fixed effects alone (marginal R^2) is of 8.92e-03. The model's intercept, corresponding to $z(openness_j) = 0$, $z(openness_i) = 0$ and $matching_{ij} = 0$, is at 2.80 (95% CI [2.65, 2.94], $t(26348) = 37.87$, $p < .001$. Within this model the effect of $z(openness_j)$ is statistically non-significant and negative (b = -5.21e-03, 95% CI [-0.14, 0.13], $t(26348) = -0.08$, $p = 0.938$; Std. β = -4.17e-03, 95% CI [-0.11, 0.10]). The effect of $z(openness_i)$ is statistically significant and positive ($b = 0.10$, 95% CI [0.04, 0.16], $t(26348) = 3.15$, $p = 0.002$; Std. β = 0.08, 95% CI [0.03, 0.13]). The effect of $matching_{ij}$ is statistically significant and negative (b = -0.07, 95% CI [-0.09, -0.05], $t(26348) = -8.00$, $p < .001$; Std. β = -0.05, 95% CI [-0.06, -0.04]).

Study 1b The model's total explanatory power is substantial (conditional $R^2 = 0.31$), and the part related to the fixed effects alone (marginal R^2) is of 8.94e-03. The model's intercept, corresponding to $z(openness_j) = 0$, $z(openness_i) = 0$ and $matching_{ij} = 0$, is at 2.73 (95% CI [2.61, 2.86], $t(48173) = 43.39$, $p < .001$. Within this model the effect of $z(openness_j)$ is statistically non-significant and positive ($b = 0.07$, 95% CI [-0.04, 0.18], $t(48173) = 1.22$, $p = 0.223$; Std. $\beta = 0.06$, 95% CI [-0.03, 0.15]). The effect of $z(openness_i)$ is statistically significant and positive ($b = 0.06$, 95% CI [9.48e-03, 0.10], $t(48173) = 2.36$, $p = 0.018$; Std. $\beta = 0.04$, 95% CI [7.62e-03, 0.08]). The effect of $matching_{ij}$ is statistically significant and negative (b = -0.08, 95% CI [-0.10, -0.07], $t(48173) = -12.86$, $p < .001$; Std. β = -0.05, 95% CI [-0.06, -0.05]).

Study 2a The model's total explanatory power is substantial (conditional $R^2 = 0.43$), and the part related to the fixed effects alone (marginal R^2) is of 0.02. The model's intercept, corresponding to $z(openness_j) = 0$, $z(openness_i) = 0$ and $matching_{ij} = 0$, is at 2.51 (95% CI [2.30, 2.72], $t(48173) = 23.59$, $p < .001$). Within this model the effect of $z(openness_j)$ is statistically non-significant and positive ($b = 0.15$, 95% CI [-0.05, 0.35], $t(48173) = 1.43$, $p = 0.152$; Std. $\beta = 0.12$, 95% CI [-0.05, 0.29]). The effect of $z(openness_i)$ is statistically non-significant and negative (b = -0.01, 95% CI [-0.06, 0.03], $t(48173) = -0.63$, $p = 0.528$; Std. β = -0.01, 95% CI [-0.05, 0.03]). The effect of $matching_{ij}$ is statistically significant and negative (b = -0.02, 95% CI [-0.03, -3.53e-03], $t(48173) = -2.52$, $p = 0.012$; Std. β = -0.01, 95% CI [-0.02, -2.38e-03]).

Study 2b The model's total explanatory power is substantial (conditional $R^2 = 0.32$), and the part related to the fixed effects alone (marginal R^2) is of 5.73e-03. The model's intercept, corresponding to $z(openness_j) = 0$, $z(openness_i) = 0$ and $matching_{ij} = 0$, is at 2.79 (95% CI [2.65, 2.94], $t(48233) = 38.03$, $p < .001$). Within this model the effect of $z(openness_j)$ is statistically non-significant and positive ($b = 0.09$, 95% CI [-0.05, 0.22], $t(48233) = 1.24$, $p = 0.214$; Std. $\beta = 0.07$, 95% CI [-0.04, 0.18]). The effect of $z(openness_i)$ is statistically non-significant and positive ($b = 0.04$, 95% CI [-6.18e-03, 0.08], $t(48233) = 1.69$, $p = 0.092$; Std. $\beta = 0.03$, 95% CI [-5.01e-03, 0.07]). The effect of $matching_{ij}$ is statistically significant and positive ($b = 0.01$, 95% CI [5.02e-04, 0.03], $t(48233) = 2.03$, $p = 0.042$; Std. β = 9.28e-03, 95% CI [3.36e-04, 0.02]).

Overall, the robustness check shows the same pattern of effects in 3/4 studies, with the exception of Study 2b.

Algorithmic validation with an open-source AI model. One possible criticism of our approach that may affect the replicability of our findings deals with the reliance on closed-source models that may soon be deprecated. To ensure our findings extend beyond a specific model, we utilized an open-source model (Meta's Llama 2 70-B) in an attempt to replicate our algorithmic validation. We followed the same prompting strategy as we did in Study 2b. We used Together AI API to access the model. The API returned valid responses to 280 pairs of ads. Consistent with the results in Study 2b, we observed a significantly higher openness score for the open ads $t(279) = 2.10$, $p = .037$, Cohen's $d = 0.13$, 95% CI [0.01, 0.24]).

References

1. JB Hirsh, SK Kang, GV Bodenhausen, Personalized persuasion: tailoring persuasive appeals to recipients' personality traits. *Psychol. Sci.* **23**, 578–581 (2012).
2. JB Nezlek, A practical guide to understanding reliability in studies of within-person variability. *J. Res. Pers.* **69**, 149–155 (2017).
3. A Simchon, A Sutton, M Edwards, S Lewandowsky, Online reading habits can reveal personality traits: towards detecting psychological microtargeting. *PNAS Nexus* **2**, gad191 (2023).
